# Serological Markers of SARS-CoV-2 Reinfection

**DOI:** 10.1128/mbio.02141-21

**Published:** 2022-01-25

**Authors:** Sameed M. Siddiqui, Kathryn A. Bowman, Alex L. Zhu, Stephanie Fischinger, Samuel Beger, Jenny S. Maron, Yannic C. Bartsch, Caroline Atyeo, Matthew J. Gorman, Ahmad Yanis, Judd F. Hultquist, Ramon Lorenzo-Redondo, Egon A. Ozer, Lacy M. Simons, Rana Talj, Danielle A. Rankin, Lindsay Chapman, Kyle Meade, Jordan Steinhart, Sean Mullane, Suzanne Siebert, Hendrik Streeck, Pardis Sabeti, Natasha Halasa, Elon R. Musk, Dan H. Barouch, Anil S. Menon, Eric J. Nilles, Douglas A. Lauffenburger, Galit Alter

**Affiliations:** a Computational and Systems Biology Program, Massachusetts Institute of Technology, Cambridge, Massachusetts, USA; b Broad Institute of MIT and Harvard, Cambridge, Massachusetts, USA; c Ragon Institute of MGH, MIT, and Harvard, Cambridge, Massachusetts, USA; d PhD Program in Immunology and Virology, University of Duisburg-Essen, Essen, Germany; e Space Exploration Technologies Corp., Hawthorne, California, USA; f PhD Program in Virology, Division of Medical Sciences, Harvard University, Boston, Massachusetts, USA; g Department of Pediatrics, Vanderbilt University Medical Center, Nashville, Tennessee, USA; h Department of Medicine, Division of Infectious Diseases, Northwestern University Feinberg School of Medicine, Chicago, Illinois, USA; i Center for Pathogen Genomics and Microbial Evolution, Institute for Global Health, Northwestern University Feinberg School of Medicine, Chicago, Illinois, USA; j Vanderbilt Epidemiology PhD Program, Vanderbilt University School of Medicine, Nashville, Tennessee, USA; k Institute of Virology, University Hospital, University of Bonn, and German Center for Infection Research (DZIF), partner site Bonn-Cologne, Cologne, Germany; l Harvard T.H. Chan School of Public Health, Cambridge, Massachusetts, USA; m Howard Hughes Medical Institute, Chevy Chase, Maryland, USA; n Massachusetts Consortium on Pathogen Readiness, Boston, Massachusetts, USA; o Center for Virology and Vaccine Research, Beth Israel Deaconess Medical Center, Harvard Medical School, Boston, Massachusetts, USA; p Brigham and Women’s Hospital, Department of Emergency Medicine, Boston, Massachusetts, USA; q Harvard Medical School, Harvard University, Cambridge, Massachusetts, USA; r Harvard Humanitarian Initiative, Boston, Massachusetts, USA; s Department of Biological Engineering, Massachusetts Institute of Technology, Cambridge, Massachusetts, USA; Duke Human Vaccine Institute; Duke University Medical Center

**Keywords:** SARS-CoV-2, reinfection, antibodies, humoral immunity, diagnostics, biomarkers

## Abstract

As public health guidelines throughout the world have relaxed in response to vaccination campaigns against SARS-CoV-2, it is likely that SARS-CoV-2 will remain endemic, fueled by the rise of more infectious SARS-CoV-2 variants. Moreover, in the setting of waning natural and vaccine immunity, reinfections have emerged across the globe, even among previously infected and vaccinated individuals. As such, the ability to detect reexposure to and reinfection by SARS-CoV-2 is a key component for global protection against this virus and, more importantly, against the potential emergence of vaccine escape mutations. Accordingly, there is a strong and continued need for the development and deployment of simple methods to detect emerging hot spots of reinfection to inform targeted pandemic response and containment, including targeted and specific deployment of vaccine booster campaigns. In this study, we identify simple, rapid immune biomarkers of reinfection in rhesus macaques, including IgG3 antibody levels against nucleocapsid and FcγR2A receptor binding activity of anti-RBD antibodies, that are recapitulated in human reinfection cases. As such, this cross-species analysis underscores the potential utility of simple antibody titers and function as price-effective and scalable markers of reinfection to provide increased resolution and resilience against new outbreaks.

## INTRODUCTION

In the setting of waning natural and vaccine-induced immunity, SARS-CoV-2 reinfections are on the rise across the globe (1–4). These new waves of infections were accompanied by accumulating reports of viral evolution and the selection of more infectious variants. Typically, reinfections have been documented by the identification of distinct viral genomic sequences in nasopharyngeal swabs collected at primary and secondary infection to differentiate authentic reinfection from transient nucleic acid positivity or persistent viral shedding. However, in the setting of declining antibody titers, reinfection has been noted even with matched strains, offering the virus an opportunity to begin to evolve around immunity. Thus, determining the immunologic markers of authentic SARS-CoV-2 reinfection, with both novel and recirculating strains, as well as the immunologic mechanism(s) associated with disease attenuation, is necessary for informed public health decisions regarding social distancing, societal reopening, vaccine development, and vaccine deployment.

Due to the transient nature of immunological memory to many human coronaviruses, the risks of reinfection are considerable (5). Given the unpredictable nature of SARS-CoV-2 disease severity and our emerging appreciation of secondary organ complications (6), there is an urgent need to define correlates of attenuated disease against SARS-CoV-2. While great effort is currently being invested into defining correlates of immunity in animal models (7), efforts to define natural correlates of infection in humans, linked to reduced severity following reinfection, may profoundly accelerate the identification of immune mechanisms involved in limiting viral replication and disease and may aid in the identification of immunologic gaps in response that may permit breakthrough infections to occur. These findings have implications for both rational vaccine design and vaccine deployment in large populations, particularly in the wake of emerging variants of concern.

The number of reinfections globally has likely been vastly underestimated, partly due to the fact that confirmation of reinfection requires identification of distinct SARS-CoV-2 strains at primary and secondary infection via viral genome sequencing. While this method is a gold standard for minimizing false positives in identifying reinfection cases, viral sequencing is technically challenging at a large scale, cannot identify cases of reinfection with the same viral strain, and provides limited insights into the immunological mechanism of antiviral control or the need to boost to prevent the spread and potential evolution of novel vaccine-escape mutations. In addition, implementation relies on sequencing capabilities not available in many areas. In the setting of these challenges, the true frequency of reinfection remains unclear. On the other hand, SARS-CoV-2 reinfection models based on historical data from seasonal coronavirus infections, recent direct evidence of declining antibody responses, and increased transmissibility of recent variant of concerns suggest the possibility of continued increases in rates of reinfection despite acquired immunity (8–11). As such, a tool to provide better resolution to the demographics of reinfection may significantly inform future health policy, including testing or focused vaccine boosting campaigns. Thus, our ability to monitor and control both infection and reinfection hinges on the development of simple, immunologically sound screening strategies capable of reliably monitoring reinfection with both novel and recirculating strains.

A rise in pathogen-specific antibody titers has been used as a biomarker of response to therapy or infection (12). Given the ease and specificity of antibody diagnostics, here we deeply profiled the changes in the humoral immune response in a tightly controlled nonhuman primate (NHP) study, where animals were infected and challenged with different inocula, allowing the identification of challenge dose-independent biomarkers of reexposure to SARS-CoV-2. Strikingly, the same immunologic signatures were validated in an individual with sequencing-confirmed reinfection and in an independent cohort of putatively reinfected humans, drawn from a large community-based serosurveillance study, that were serologically positive with a subsequent PCR-positive test. Here, we identify a minimal set of SARS-CoV-2-specific markers of reinfection with robust discriminatory cross-validating power across both primates and humans. Thus, simple serological analytes may support the identification of SARS-CoV-2 reinfections at a global level.

## RESULTS

### Rhesus macaque SARS-CoV-2 reinfection is associated with robust antibody boosting.

**FIG 1:**
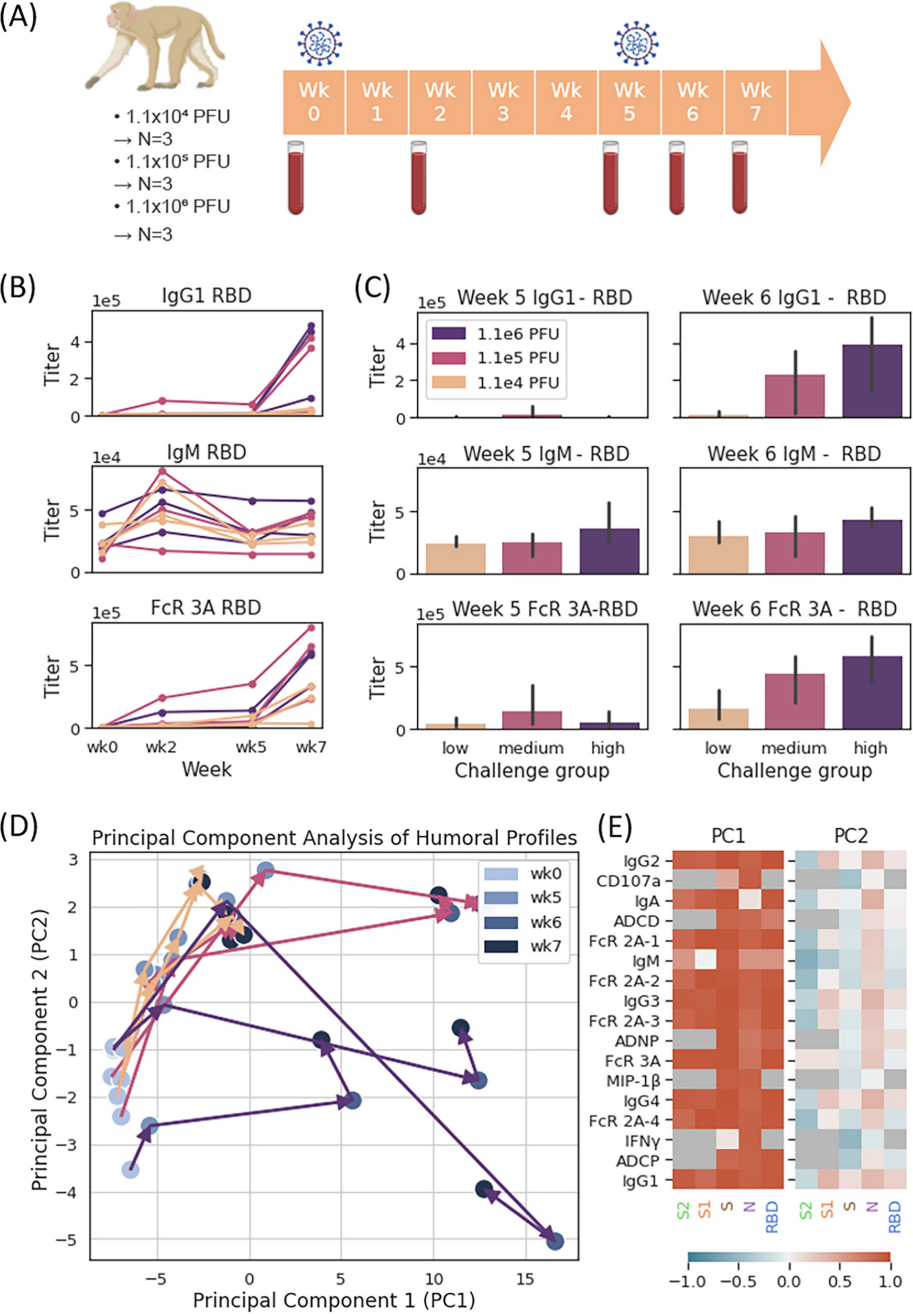
Immune response to primary infection and reexposure in rhesus macaques. (A) Pictogram of rhesus macaque study design. Nine rhesus macaques were challenged on week 0 and week 5, with sample collections on week 0 (prior to first challenge), week 2, week 5 (prior to rechallenge), week 6, and week 7. (B) IgG1, IgM, and FcR3A binding titers to RBD antigen as a function of week, categorized by challenge and rechallenge titer (beige, 1.1e4 PFU; mauve, 1.1e5 PFU; purple, 1.1e6 PFU). (C) IgG1, IgM, and FcR3A binding titers to RBD antigen in week 5 (primary infection) and week 6 (reinfection). A two-sided Mann-Whitney *U* test was used to calculate *P* values comparing response in the low-challenge groups versus that in the medium- and high-challenge groups. After multiple-hypothesis correction, no significant differences were found. (D) Principal-component analysis (PCA) plot of monkey trajectories, with week indicated by color and challenge group indicated by arrow color (beige arrows, low challenge; mauve arrows, medium challenge; indigo arrows, high challenge). We note that the color gradient of markers from light to dark reflects the timeline, with serum samples at week 0 marked by light blue circles and samples at week 7 marked by dark blue circles. (E) Principal-component analysis loading heatmap in rhesus macaques, with 77.7% and 6.1% of variance explained by PC1 and PC2. Feature loadings represented by color from dark blue (loading = −1) to dark red (loading = +1), with features not collected for analysis colored in dark gray.

Mounting evidence points to the utility of SARS-CoV-2 infection in rhesus macaques as an informative model for human infection, with an observable spectrum of clinical disease severity following infection accompanied by striking pathological similarities to those observed in humans deep within the lungs (13). In a previous study to define whether primary SARS-CoV-2 challenge confers protection upon rechallenge, 9 adult rhesus macaques were challenged and rechallenged with SARS-CoV-2 (14). Macaques were challenged with 1.1 × 10^6^ PFU (high dose, *N =* 3), 1.1 × 10^5^ PFU (medium dose, *N =* 3), and 1.1 × 10^4^ PFU (low dose, *N =* 3) administered intranasally and intratracheally. At week 5, the same dosages were used for rechallenge as the initial week 0 challenge (Fig. 1A). Importantly, while animals exhibited some viral replication in nasal swabs, they exhibited marginal viral replication in the lungs and little to no lung pathology following rechallenge, suggesting that primary infection elicits protective immunity for at least 1 month. However, critically, rechallenge was performed with the same strain of SARS-CoV-2, and, as such, although viral replication was detectable in the nasal tract, viral sequencing would not in this case have been able to define reinfection. Using this highly controlled study, we aimed to determine whether virus-specific immunological biomarkers could be defined to profile the response to rechallenge.

While the previous study noted the induction of humoral immunity across all animals 5 weeks after primary infection (14), here we comprehensively profiled the humoral immune response before and after both primary challenge and rechallenge. Low but positive IgG responses were observed in all animals following primary infection (week 5) (Fig. 1B). However, following reinfection, a dose-dependent increase in SARS-Cov-2-specific IgG was observed (week 7) (Fig. 1C), with significantly higher responses in the medium- and high-dose rechallenge groups than in the low-dose rechallenge group. IgM responses increased as expected at the primary response and remained largely stable or slightly increased at rechallenge (Fig. 1B and C). Conversely, Fc receptor binding activity of SARS-Cov-2-specific antibodies increased significantly after primary infection and also increased significantly across all animal groups following rechallenge (Fig. 1B and C). Notably, the increase in Fc receptor binding was more pronounced at rechallenge (Fig. 1C), even in the low-dose challenge, suggesting that qualitative changes in the inflammatory profile of SARS-CoV-2-specific IgG represent a highly sensitive biomarker of viral reexposure to the virus.

**FIG 2:**
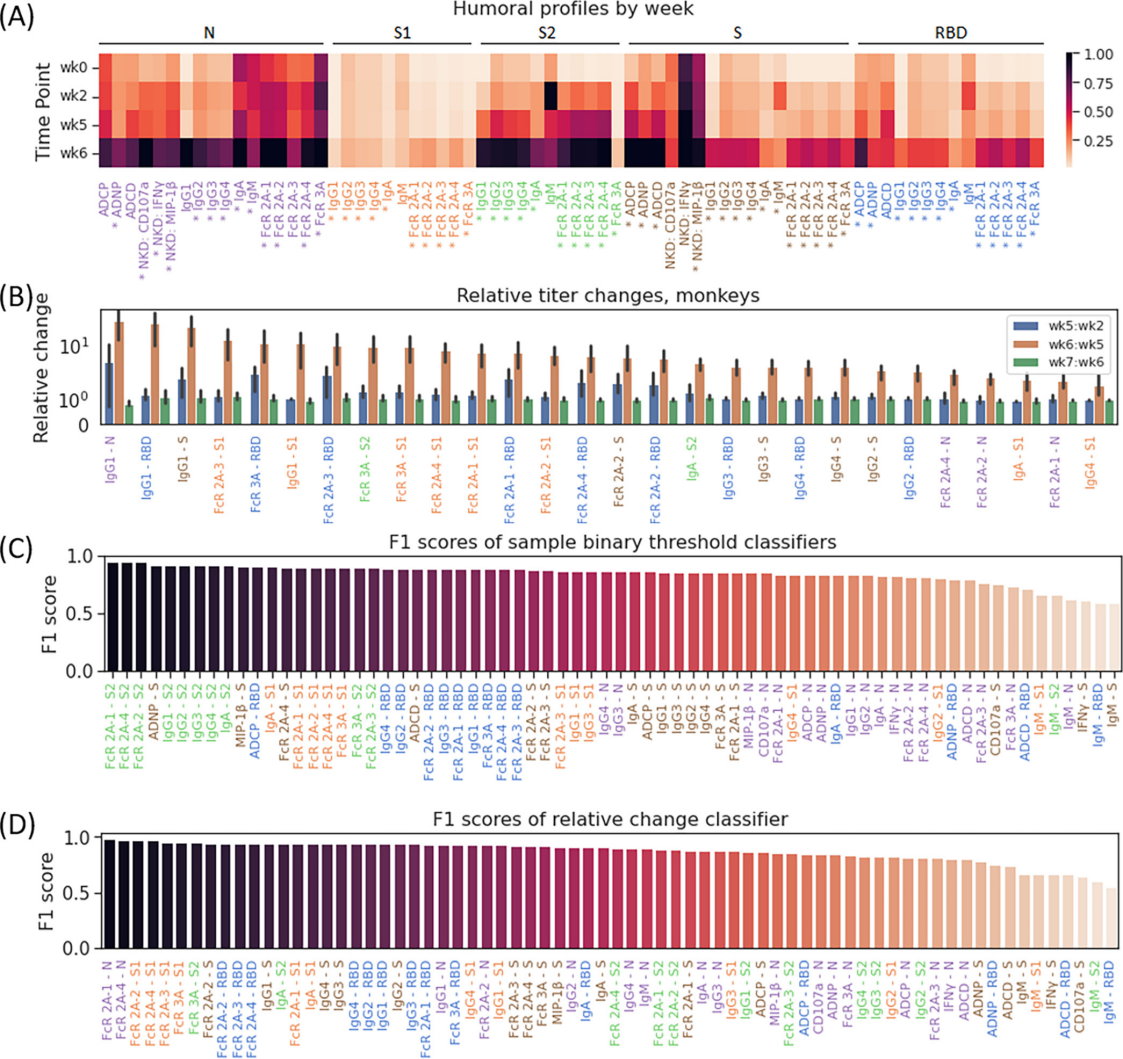
Markers associated with reexposure in rhesus macaques. (A) Heatmap of collected Luminex and functional features across weeks 0, 2, 5, and 6. An asterisk indicates differential expression between week 5 and week 6 with a false discovery rate of 5%. (B) Per-sample change in relative titer of the 25 features with maximal relative change in macaques between primary infection (week 5) and reinfection (week 6). (C and D) F1 scores of all sample binary threshold classifiers (C) and relative change-based binary classifiers (D) in rhesus macaques, with labels colored by antigen.

To examine the trajectory of the overall humoral immune response in an unbiased manner, the multivariate trajectory of the humoral immune response was profiled across all animals. Interestingly, despite differences in the magnitude of the response across different dosage groups, similar trajectories were observed across all animals (Fig. 1D). Notably, all animals exhibited increases in both principal component 1 and principal component 2 (PC2), together marking primary infection increases in acute IgG3, IgG1, Fcγ-receptor binding, and functional humoral immunity (Fig. 1E; see also Fig. S1A in the supplemental material), although these changes during primary infection were more attenuated than those observed at rechallenge (Fig. 1D and E). Upon reinfection, a burst of functional and humoral immunity was observed across all animals, including subclass, isotype, and Fc-receptor binding antibodies, with notable bursts in IgG1 titers to SARS-CoV-2 broadly across nucleocapsid (N) and spike (S) (Fig. 1E). More variable but consistent increases were also observed for the spike receptor binding domain (RBD) and the S1 domain of spike, with FcγR2A-3 and FcγR3A binding to RBD, S, and S1 exhibiting the largest mean functional increase (Fig. 2A and Fig. S1A), highlighting particular specificities and FcR binding profiles as the best discriminators/indicators of rechallenge. These data point to significant changes in antibody boosting following reinfection, suggesting a potential utility of serological boosting as an antigen-specific biomarker of reinfection.

10.1128/mBio.02141-21.1 FIG S1 Principal-component analysis loading plots in rhesus macaques, with 77.7% and 6.1% of variance explained by PC1 and PC2, respectively (A), and humans, with 49.1% and 12.2% of variance explained by PC1 and PC2, respectively (B). We note that panel A is a bar plot representation of the information conveyed via heatmap in Fig. 1E. Download FIG S1, EPS file, 1.1 MB. Copyright © 2022 Siddiqui et al. 2022 Siddiqui et al. https://creativecommons.org/licenses/by/4.0/ This content is distributed under the terms of the Creative Commons Attribution 4.0 International license.

### Defining minimal markers of reinfection in the rhesus macaque model of SARS-CoV-2 infection.

To further track the kinetics of humoral evolution across the individual features, we compared the humoral immune response across the S, RBD, S1, S2 domain of spike, and N (Fig. 2A). A striking evolution of S, RBD, S2, and N-specific immunity was observed across all animals, most dramatically in IgG1 titer to spike, RBD, and nucleocapsid and FcγR2A receptor binding to S1, spike, and RBD, with mean increases of 31.3-fold and 7.6-fold, respectively, upon reinfection (Fig. 2B, Fig. S3A). Notably, increased functional activity in parallel to the robust induction of Fc receptor binding antibodies was also observed, with average increases of 2.1-fold, 1.7-fold, and 2.6-fold upon rechallenge in antibody-dependent cellular phagocytosis (ADCP), antibody-dependent complement deposition (ADCD), and antibody-dependent neutrophil phagocytosis (ADNP).

10.1128/mBio.02141-21.3 FIG S3 Plot of anti-RBD IgG1 and anti-RBD FcR2A-1 titer in each macaque time point (A) and of anti-RBD IgG1 and anti-RBD FcR2A titer in each human time point (B), highlighting reinfection-associated changes in titer. Download FIG S3, EPS file, 0.3 MB. Copyright © 2022 Siddiqui et al. 2022 Siddiqui et al. https://creativecommons.org/licenses/by/4.0/ This content is distributed under the terms of the Creative Commons Attribution 4.0 International license.

Due to the consistencies in principal-component analysis (PCA) trajectories and notable changes in titer upon reexposure, we hypothesized that certain immune features or trends could be used to identify reinfection or reexposure in primates. First, we identified simple binary thresholds that could be used to identify individual samples as primary infection or as reexposure. We observed that levels of FcγR2A-1, Fcγ2A-2, and Fcγ2A-3 binding antibodies to S2 and IgG1, IgG2, IgG3, and IgG4 to S2 served as strong binary thresholds to identify individual samples, with true-positive rates of 1.0 and 1.0, false-positive rates of 0.11 and 0.17, f_1_ scores of 0.95 and 0.92, and classification accuracies of 94% and 91% in the FcR and IgG classifiers, respectively (Fig. 2C and Table S1). Binary thresholds to many other features also performed well, with more than half of all features, including all anti-RBD IgG titers and all anti-RBD FcR binding titers, performing with an f_1_ score of >0.85 and classification accuracy of >86%. However, we noted that IgM antibodies were consistently the worst set of predictors, with an average f_1_ score of 0.63 and an average classification accuracy of 59% among the different targeted antigens.

10.1128/mBio.02141-21.5 TABLE S1 Binary threshold-based classifiers in rhesus macaques, based on titer value from one serum sample. Download Table S1, XLSX file, 0.01 MB. Copyright © 2022 Siddiqui et al. 2022 Siddiqui et al. https://creativecommons.org/licenses/by/4.0/ This content is distributed under the terms of the Creative Commons Attribution 4.0 International license.

While the performance of most of these thresholds is promising, models accounting for immune profile changes across time points may confer an additional degree of robustness to various immune responses, specifically as noted across various inoculum sizes (Fig. 1A). As such, we created simple univariate classifiers to determine if the difference in macaque immune response between any two collected time points was associated with reinfection or simply reflected a continued response to initial exposure (Fig. 2D, Table S2). Interestingly, while a majority of features performed very well (f_1_ score of >0.90, classification accuracy of >88%), the 8 best features were all Fc-receptor binding quantities, performing with f_1_ scores above 0.94 and classification accuracy of >92%. Among antibody titer-based predictors, IgGs to Spike, IgA to S1 and S2, and IgG1, IgG2, IgG3, and IgG4 to RBD served as the best predictors, with each predictor correctly differentiating 92% of all pairs of responses (f_1_ score, 0.94) as reexposure or continued response with thresholds of 3.3, 1.4, 1.6, and 1.6 for changes in anti-RBD IgG1, IgG2, IgG3, and IgG4 titer, respectively. While classifiers using nucleocapsid responses were slightly less effective, their thresholds (1.2 for IgG1, 1.1 for IgG2, 1.7 for IgG3, and 1.2 for IgG4) were similar to the 1.4 ratio for IgG-N used by Edridge et al. for detection of reinfection by other coronaviruses (15). Collectively, these data demonstrate a clear and predictable increase in the expression of a broad range of humoral features upon reinfection in rhesus macaques, suggesting a biomarker-based approach to identifying reinfection in humans.

10.1128/mBio.02141-21.6 TABLE S2 Binary threshold-based classifiers in rhesus macaques, based on relative change in titer between chronologically paired serum samples. Download Table S2, XLSX file, 0.01 MB. Copyright © 2022 Siddiqui et al. 2022 Siddiqui et al. https://creativecommons.org/licenses/by/4.0/ This content is distributed under the terms of the Creative Commons Attribution 4.0 International license.

### Antibody profiles following reinfection in humans.

**FIG 3:**
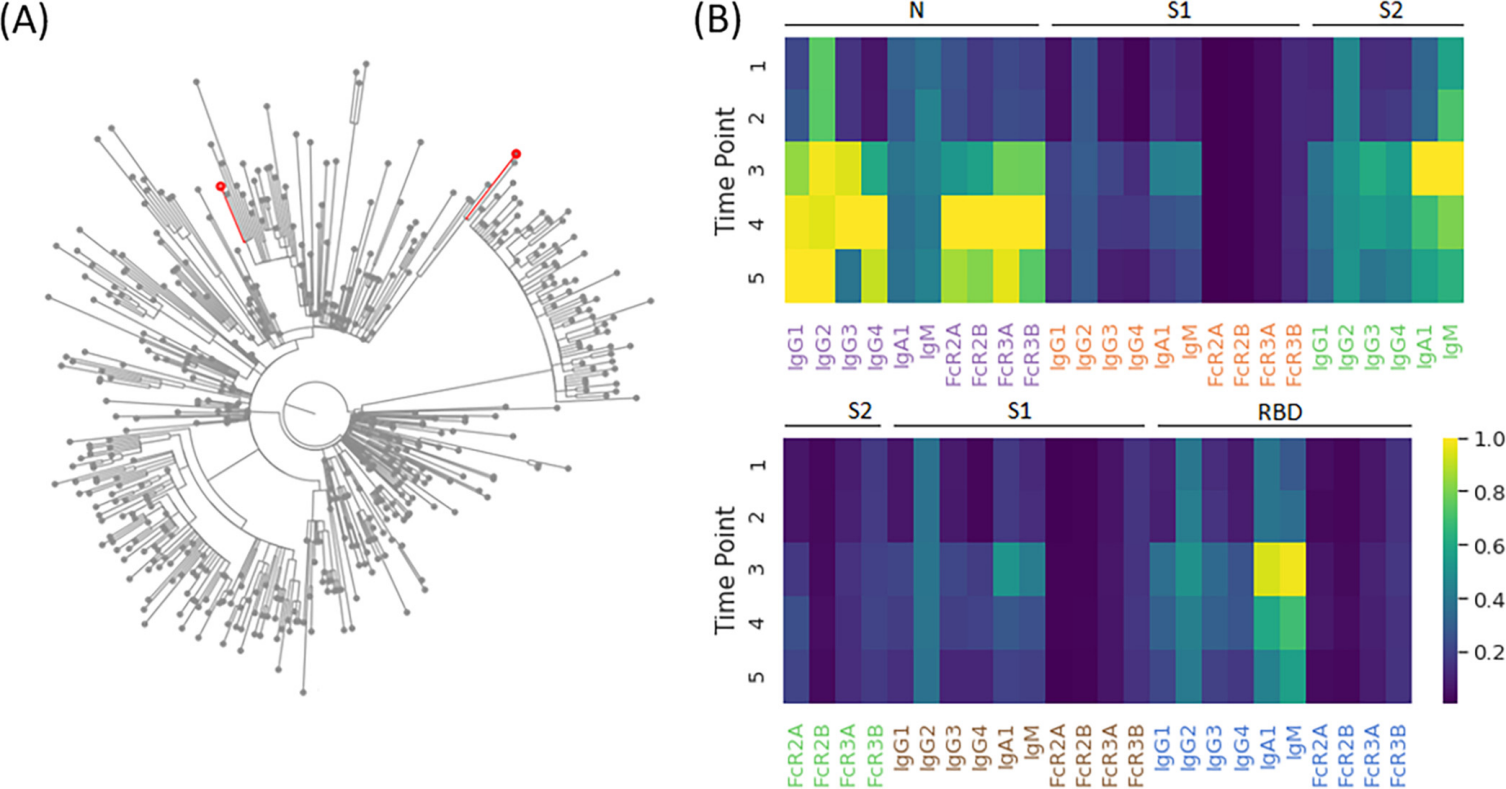
Immune response to sequencing-confirmed reinfection. (A) Maximal likelihood phylogenetic tree with 500 global sequences randomly sampled from GISAID, with patient viral lineage B.1.2 and B.1.429 demarcated with red nodes. (B) Heatmap of collected Luminex and functional features across the respective time points in the patient, corresponding to 34, 70, 126, 159, and 208 days after onset of symptoms during primary infection. Of note, reinfection was identified and confirmed with sequencing between time points 2 and 3.

Like primates, which were recolonized in the upper respiratory tract by the same viral strain (Fig. 1), the rise of reinfections globally clearly highlights the susceptibility in humans to emerging variants (11, 16–18). In the setting of waning immunity and emergence of new variants, reinfections are on a dramatic rise globally (10, 16, 17, 19). To begin to examine whether reinfection in humans is also associated with specific humoral changes, we performed humoral immune profiling of longitudinal serum samples from 3 individuals with suspected reinfection based on recurrent PCR positivity with recurrent symptoms, greater than 45 days from initial date of positive PCR, per CDC investigative guidance (20). Concurrent viral sequencing from nasopharyngeal swabs at the time of initial symptom onset and subsequent symptom onset with repeat PCR positivity identified one individual with sequence-confirmed SARS-CoV-2 reinfection by a distinct viral lineage. Virus sampled at the time of initial infection belonged to the B.1.2 lineage, the current dominant lineage in the United States for some time. The second virus, collected 85 days after initial swab, belonged to B.1.429, one of the initial lineages of concern originally found in California (Fig. 3A). Longitudinal biophysical antibody profiling demonstrated increased titers of all antibody titers previously tested except IgM to nucleocapsid, recapitulating the observed patterns of reinfection in the rhesus macaques (Fig. 3B). Notably, the largest increases in titer in this patient were of IgG4 against nucleocapsid, spike, and S1, with increases of 8.56-, 8.46-, and 7.30-fold, respectively, and of IgG3 against nucleocapsid, which increased by a factor of 6.16.

**FIG 4:**
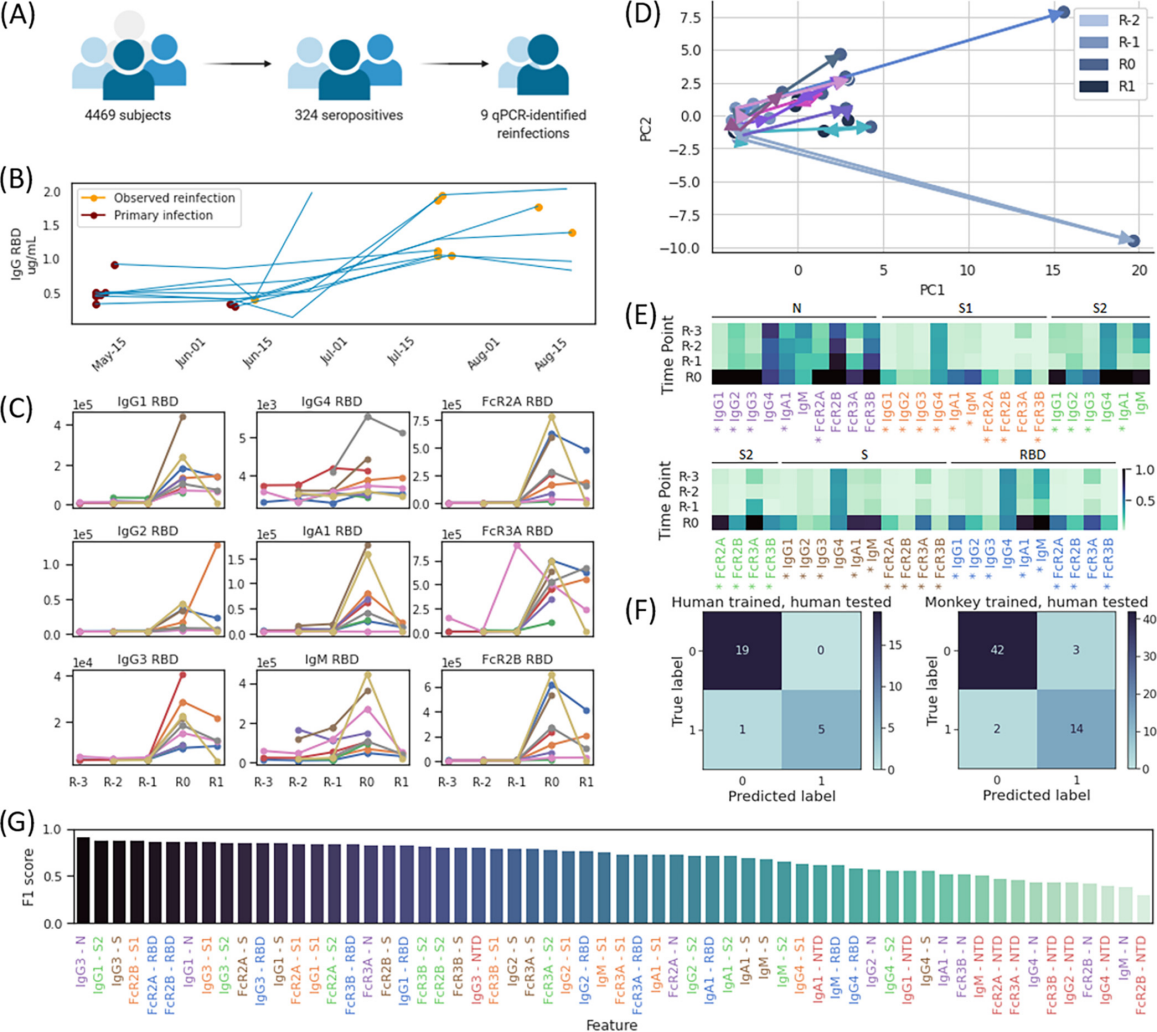
Immune response to and discriminating biomarkers for primary infection and reinfection in humans. Cases identified through a community-based surveillance survey with R0 defined as the serum sample associated most closely with time of putative reinfection, R-1, R-2, and R-3, defined as the first, second, and third serum samples preceding reinfection, and R1, defined as the serum sample immediately after R0; for each subject, the earliest included time point is the first recorded seropositive sample. (A) Pictogram of community-based serological surveillance. (B) ELISA titers to IgG RBD in each PCR-confirmed subject collected at different time points between 12 May 2020 and 19 August 2020, with time points of first recorded seropositivity and observed reinfection denoted by purple and gold markers, respectively. (C) IgG1, Ig2, IgG3, IgG4, IgGA1, IgM, FcR2A binding, FcR3A binding, and FcR2B binding titers to RBD antigen as a function of collected time point in each subject. (D) Principal-component analysis (PCA) plot of human trajectories, with trajectories of different subjects indicated with differently colored arrows. We note that the color gradient of markers from light blue to dark blue reflects the timeline, with serum samples at R-2 marked by light blue circles and samples at R1 marked by dark blue circles. (E) Heatmap of collected Luminex and functional features across the respective time points in each subject. An asterisk indicates differential expression between week R-1 and R0 with a false discovery rate of 5%. (F) Confusion matrix of two-feature logistic regression models trained and tested in humans (left) and trained in rhesus macaques and tested in humans (right). (G) F1 scores of all relative change-based binary classifiers trained and tested in humans, with labels colored by antigen.

To further characterize transmission and infection of SARS-CoV-2 outside hospital settings, a community-based serosurveillance cohort was established at Space Exploration Technologies Corp. (SpaceX) as previously described (21). As a part of this program, regular antibody- and PCR-based follow-up was conducted on a cohort of 4,469 volunteers since May 2020, with 2,130 volunteers participating in serum collection at least twice, with a mean time of approximately 39 days between sample collections. This led to the identification of 324 seropositive individuals by November 2020 (Fig. 4A). However, 9 individuals that were persistently seropositive became PCR^+^ 15 to 55 days (mean 39 days) following a seropositive test result. While viral sequencing was not available in this study, we aimed to examine whether this renewed PCR^+^ result was evidence of potential reinfection.

In the absence of the viral sequence, we aimed to determine whether similar increases in antibodies, compared to the animal model, were observed in these 9 individuals, marking potential reexposure. Antibody titers increased in eight out of nine individuals after the positive PCR test with a mean change of 3.0-fold increase in titer (Fig. 4B), indicating an antibody boost in humans similar to observations in primates (Fig. 1). To assess whether these 9 PCR-positive samples were cases of authentic reinfection/reexposure, comprehensive antibody profiling was performed. As observed in the primates, antibody responses were low early in the study, although all individuals were antibody positive per our highly specific RBD-specific antibody enzyme-linked immunosorbent assay (ELISA) (22). Importantly, in a general cohort of 31 seropositive individuals who remained PCR negative over the study period, and, thus, were not suspected to be reinfected, we noted more limited changes in ELISA titer and distinct differences in patterns of antibody profile in the general cohort compared to the reinfection cohort (Fig. S4).

10.1128/mBio.02141-21.4 FIG S4 (A) Comparison of ELISA titer increases in the general (*N* = 31) and PCR-identified reinfections (*N* = 9). Eight out of 9 samples in the reinfection cohort increased in ELISA titer between primary and reinfection by more than 1.2× compared to 7 out of 31 samples in the general cohort. (B to F) Antibody profiles in the reinfection cohort and the 7 general cohort samples with ELISA titer change of >1.2×. Titers in the reinfection cohort (colored from cyan to purple between time points R-3 and R1) are seen to increase at the time point of identified infection, whereas in the general cohort (colored from red to green), only one sample (GC2) displays an antibody profile similar to that of reinfected individuals. We note that this individual did not volunteer for a PCR test, and, as such, their reinfection status could not be verified. Download FIG S4, EPS file, 0.5 MB. Copyright © 2022 Siddiqui et al. 2022 Siddiqui et al. https://creativecommons.org/licenses/by/4.0/ This content is distributed under the terms of the Creative Commons Attribution 4.0 International license.

As observed in macaques, antibody titers for most features increased upon reinfection, with different amounts of change observed across individuals (Fig. 4C). A significant increase in antibody levels was noted across antibodies and functions to N, S1, S2, S, and RBD, but a more limited increase to IgG titers in the N-terminal domain of Spike (NTD) was observed across the cohort (Fig. 4E). Notably, while titers of IgM to N decreased upon reinfection in most individuals with a mean change of −11%, levels of IgM to NTD, RBD, S2, S, and S1 rose in most individuals by mean fold changes of 2.2, 5.1, 5.6, 12.8, and 13.8.

Interestingly, as seen in macaques, multivariate profiles highlighted the same directional increase in antibody quality across principal component 1 (PC1) in all of the potentially reinfected individuals (Fig. 4D, Fig. S1B); these changes included increases in titer and function selectively upon reinfection (Fig. S2B and S3B), again supporting the identification of predictors to identify reinfection based on antibody profile. When testing humoral features as predictors on a combined cohort of the 9 putative reinfection subjects and of the 31 seropositive, non-reinfection-identified subjects, we found that simple binary thresholds of univariate features were not sufficient to produce robust classifiers of reinfection, perhaps due to varied immune responses and inoculum in the individuals. However, several models comparing the relative difference of titer between subsequent samples were effective, with 24 out of 60 features performing with f_1_ scores of ≥0.80 and classification accuracies above 89% (Fig. 4G, Table S3). Interestingly, IgG1 and IgG3 antibodies to RBD, S, S1, S2, and N were the most effective antibody predictors, all with f_1_ scores of >0.83 and classification accuracies of >90%, whereas 9 out of 10 NTD-based features were among the 20 worst predictors, performing with a mean f score of 0.49 and a mean classification accuracy of 60%.

10.1128/mBio.02141-21.2 FIG S2 Per-sample change in relative titer of the 25 features with maximal relative change in humans between the serum sample closest to identified reinfection and its immediately preceding sample. Download FIG S2, EPS file, 0.6 MB. Copyright © 2022 Siddiqui et al. 2022 Siddiqui et al. https://creativecommons.org/licenses/by/4.0/ This content is distributed under the terms of the Creative Commons Attribution 4.0 International license.

10.1128/mBio.02141-21.7 TABLE S3 Binary threshold-based classifiers in humans, based on relative change in titer between chronologically paired serum samples. Overall cohort is drawn from 9 PCR-identified reinfection cases and from 31 general cases. Download Table S3, XLSX file, 0.01 MB. Copyright © 2022 Siddiqui et al. 2022 Siddiqui et al. https://creativecommons.org/licenses/by/4.0/ This content is distributed under the terms of the Creative Commons Attribution 4.0 International license.

We next generated multivariate models by training logistic regression models to identify reinfection given the relative difference in titer across two time points of any two features. As before, we observed a variety of effective models: out of the 50 top models chosen post-cross validation (comparing 780 total models trained), 49 performed with f_1_ scores of ≥0.80 and classification accuracies of ≥92%, with 39 models including IgG3 antibody titer against nucleocapsid (Table S4). The top 14 models performed equally, with identical confusion matrices, f_1_ scores of 0.91, and classification accuracies of 96% (Fig. 4F).

10.1128/mBio.02141-21.8 TABLE S4 Logistic regression classifiers trained and tested on humans, based on relative change in titer between chronologically paired serum samples of two humoral features. Models were trained and cross-validated by 4-fold cross validation and split 80/20 for training/cross validation and testing. Confusion matrix is displayed as [true negative, false positive, false negative, true positive]. Download Table S4, XLSX file, 0.01 MB. Copyright © 2022 Siddiqui et al. 2022 Siddiqui et al. https://creativecommons.org/licenses/by/4.0/ This content is distributed under the terms of the Creative Commons Attribution 4.0 International license.

To examine the human putative reinfection findings based on the controlled monkey reinfection data, we trained logistic regression models on primate data and tested these against our human cohort. Specifically, we trained two-feature models on the relative change in titer between time points in rhesus macaques, performed cross validation on these models using our human data, and tested the top 50 resulting models in a holdout test set in humans. Interestingly, out of these 50 final models, 43 models included one immunoglobulin titer feature and one Fcγ-binding titer feature, 39 models included Fcγ-2A binding to Spike RBD, and 22 models included an IgG3 antibody (Table S5). As in the human-trained models, the top monkey-trained models performed equally, with the top 46 models having identical confusion matrices, f_1_ scores of 0.85, and classification accuracies of 92% (Fig. 4F), demonstrating the direct applicability of the primate reinfection signatures on suspected human reinfections.

10.1128/mBio.02141-21.9 TABLE S5 Logistic regression classifiers trained in macaques and directly tested in humans, based on relative change in titer between chronologically paired serum samples of two humoral features. Models were trained in monkeys, applied in humans with a 50/50 split between cross validation and testing sets. Confusion matrix is displayed as [true negative, false positive, false negative, true positive]. Download Table S5, XLSX file, 0.01 MB. Copyright © 2022 Siddiqui et al. 2022 Siddiqui et al. https://creativecommons.org/licenses/by/4.0/ This content is distributed under the terms of the Creative Commons Attribution 4.0 International license.

Collectively, using a highly controlled NHP model of reinfection coupled to a large serosurveillance study in industry workers, we demonstrate a specific set of SARS-CoV-2-specific humoral features, including spike RBD-specific IgG1 titers, nucleocapsid-specific IgG3 titers, and anti-spike RBD FcγR2A binding activity, as robust biomarkers of reinfection that translate across species. Notably, while models created using these features perform well to identify reinfection when tested in the same species, the models also perform well to identify reinfection in humans even when trained on patterns of reinfection in NHP, pointing to the highly conserved nature of these humoral changes upon reexposure to virus. Crucially, these patterns of immune boosting were also observed in a sequencing-confirmed case of reinfection in a human patient. Thus, when reinfection cannot be confirmed by viral sequence, due to limited access to the technology or due to limited variation of circulating strains within a geographic region, changes in SARS-CoV-2 humoral immune responses may offer inexpensive, reliable, and effective measures to track reinfection with SARS-CoV-2.

## DISCUSSION

Concerns over the durability of the immune response to SARS-CoV-2 have emerged in tandem with emerging viral variants (17, 19, 23) and cases of reinfection (3, 9, 17, 19, 23). For other common coronaviruses, immunologic memory appears to be transient, with rapidly declining antibody titers over just a few months (24, 25). Moreover, significant heterogeneity has been noted in antibody levels across convalescent populations (26), with the highest levels of antibodies noted in cases of greatest disease severity (27), pointing to the possibility that not all convalescents are equally protected following the resolution of infection. Whether differences in antibody levels or waning immunity renders individuals vulnerable to reinfection remains unclear, but the development of simple biomarkers able to identify reexposure or reinfection could dramatically improve our ability to identify susceptible individuals and to adjust our public health response accordingly.

In the absence of reinfection with a novel SARS-CoV-2 strain, sequence-based diagnosis of reinfection will be difficult. However, with waning vaccine immunity (28, 29), variants such as the delta variant may continue to cause new cycles of reinfections across the globe, providing fertile ground for subvariants to emerge. Because PCR testing is not available globally and lower-sensitivity antigen testing may miss cases of reinfection, the development of tools able to rapidly identify clusters of reinfections may guide the identification of novel variants caused by viruses able to circumvent vaccine-induced immunity. The need to contain these vaccine-escape variants is critical.

Because of the strikingly heterogeneous levels of antibodies that evolve following infection, longitudinal observations of fold increases in antibody titers have shown limited promise in the identification of potential reinfections, as serial serum samples often are not available. Thus, defining the immunologic signatures of reinfection provides an ancillary axis that, in addition to more expensive viral genetic sequencing, provides a simple approach to prospectively surveil for the presence of reinfection in a community. From a public health perspective, the use of humoral biomarkers offers advantages of scalability and, unlike viral sequencing, is not limited in cases of reinfection with the same viral strain. Ultimately, owing to inevitable variability in appropriate sample and resource availability, adoption of a multipronged approach using a mix of clinical data, viral sequencing, and humoral signatures of reinfection will strengthen our ability to identify reinfections rapidly, with the potential to identify hot spots of reinfection associated with new and emerging variants of concern. Moreover, it is plausible that while we are unable to define a precise window of time when a serologic approach can effectively identify reinfection, more frequent sampling in the future may define precise FcR binding/IgG level ratios that may even point to timing of reinfection. Thus, further studies on the kinetics of these immunologic signatures of reinfection will be helpful to further refine and expand the utility of these markers for maximal public health impact.

While limited reinfections were observed early in the pandemic (8, 30), reinfections are on the rise in the setting of variants of concern (10, 16) and waning immunity (28, 29, 31, 32), resulting in waves of viral evolution in both previously naturally immune and vaccinated populations (3, 33–35). Reinfections have been linked to a wide range of symptom profiles, ranging from asymptomatic infection to severe disease/hospitalization (3, 18, 26). Thus, here we aimed to use this robust and highly controlled animal challenge model, capturing even mild cases of reinfection. The use of this animal model coupled to a large serosurveillance study allowed us to determine whether non-sequence-based biomarkers exist that can detect reexposure/reinfection. We observed a clear rise in SARS-CoV-2 antibodies across all rechallenged animals with a dose-dependent rise in titers, most significantly of IgG1s and of Fc-receptor binding. Moreover, human data from 9 seropositive individuals that developed a PCR-positive test largely mirrored primate antibody changes, highlighting the conservation of antibody biomarkers of reinfection across species.

Our data showed that models using IgG differences alone performed comparably to models using antibody binding differences to Fc receptors in both primates and humans. However, the increases in Fc receptor binding activity following reinfection were found to be generally larger in magnitude in humans and were less challenge dose-dependent in macaques than IgG titers, pointing to the utility of these qualitative changes on antigen-specific antibodies as more sensitive biomarkers of reexposure. This disconnect between titers and Fc receptor binding relates to the difference in quantity and quality of antibodies that are induced following infection and rechallenge, where the inflammatory state of an antibody often increases disproportionately to the titer under inflammatory conditions. This reinforces the paradigm that, soon after rechallenge, copious amounts of antibodies, with enhanced functions, are generated to rapidly clear pathogens (36, 37). This early production of more functional antibodies is generated by large numbers of expanding plasmablasts, our body’s antibody factories, poised to respond within days of infection and drive a rapid rise of protective antibodies. Recent data suggest that the detection of highly functional antigen-specific antibodies can predict autoimmune flares (38), tumor relapse (39), and infectious disease progression (40). Likewise, the inclusion of metrics that can pick up both quantitative and qualitative alterations in SARS-CoV-2 antibodies may provide a more sensitive, holistic, and perhaps earlier marker of SARS-CoV-2 reinfection.

As evidence of waning immunity accumulates and the numbers of breakthrough infections and reinfections continue to accrue globally in highly vaccinated and immune populations (9, 10, 16), the need for boosting in specific populations has become evident. However, in the absence of a threshold of antibodies that predict protection, the need to monitor for reinfection is likely to be key to guiding future boosting timelines and to identify clusters of vaccine breakthrough infections to prevent evolution of the virus to evade vaccine-induced immunity. As such, the ability to detect both symptomatic and asymptomatic reinfections, even with genetically matched strains, is likely to be key to identifying clusters of reinfections, providing information on specific vulnerable populations as well as an opportunity to prevent evolution around immunity. An increased emphasis on serology-based diagnostics can help address this problem, providing tools to rapidly monitor the spread and trajectory of the epidemic across large populations of individuals potentially reexposed to recirculating strains. As reinfection complicates the trajectory of this pandemic, a shift in diagnostic practices implemented in conjunction with the findings of this study can offer critical insights both in defining immunological hallmarks of reinfection caused by this unpredictable and highly infectious virus and in reducing its further spread by identifying areas for targeted pandemic response, characterized by high rates of reinfection.

## MATERIALS AND METHODS

### Luminex isotype and FcR binding assay.

To determine relative concentrations of antigen-specific antibody isotypes and Fc receptor binding activity, a Luminex isotype assay was performed (41). Antigens (SARS-CoV-2 spike, RBD, N, S1, and S2) (note antigen sources) were covalently coupled to different Luminex microplex carboxylated bead regions (Luminex Corporation) using *N*-hydroxysuccinimide (NHS)-ester linkages by utilizing 1-ethyl-3-(3-dimethylaminopropyl)carbodiimide hydrochloride (EDC) and NHS (Thermo Scientific) according to the manufacturer’s recommendations. Immune complexes were formed by incubating antigen-coupled beads with diluted samples. Mouse-anti-rhesus antibody detectors were then added for each antibody isotype (IgG1, IgG2, IgG3, IgG4, and IgA; NIH Nonhuman Primate Reagent Resource, supported by AI126683 and OD010976). Tertiary anti-mouse-IgG detector antibodies conjugated to phycoerythrin (PE) were then added. FcR binding was quantified similarly by using recombinant NHP FcRs (FcγR2A-1, FcγR2A-2, and FcγR3A; courtesy of Duke Protein Production Facility) conjugated to PE as secondary detectors. Flow cytometry was performed using a 3-laser BD LSR II flow cytometer. Analysis of the flow cytometry data was performed using FlowJo software.

### System serology.

To quantify antibody functionality of plasma samples, bead-based assays were used to measure antibody-dependent cellular phagocytosis (ADCP), antibody-dependent neutrophil phagocytosis (ADNP), and antibody-dependent complement deposition (ADCD), as previously described (42). Protein antigens included prefusion stabilized spike ectodomain (S; courtesy of Bing Chen, Children’s Hospital and MassCPR), SARS-CoV2 receptor binding domain (RBD; courtesy of Aaron Schmidt, Ragon Institute and MassCPR), and nucleocapsid (N; Aalto Bio Reagents). Biotinylated antigens S, RBD, and N were coupled to fluorescent streptavidin beads (Thermo Fisher) and incubated with plasma samples to allow antibody binding to occur. For ADCP, cultured human monocytes (THP-1 cell line) were incubated with immune complexes, during which phagocytosis occurred. For ADNP, primary neutrophils were isolated from whole blood using an ammonium-chloride-potassium (ACK) lysis buffer. After phagocytosis of immune complexes, neutrophils were stained with an anti-CD66b Pacific Blue detection antibody (BioLegend) prior to flow cytometry. For ADCD, lyophilized guinea pig complement (Cedarlane) was reconstituted according to the manufacturer’s instructions and diluted in a gelatin veronal buffer with calcium and magnesium (Boston BioProducts). After antibody-dependent complement deposition occurred, C3 bound to immune complexes was detected with fluorescein isothiocyanate (FITC)-conjugated goat IgG fraction to guinea pig complement C3 (MP Biomedicals). For quantification of antibody-dependent NK cell activation, diluted plasma samples were incubated in ELISA plates coated with antigen. Human NK cells were isolated the evening before from healthy buffy coat donors using RosetteSep (STEMCELL Technologies) and incubated overnight with human recombinant interleukin-15 (STEMCELL Technologies). NK cells were incubated with immune complexes and then stained with CD107a PE-Cy5 (BD), Golgi stop (BD), and brefeldin A (BFA; Sigma-Aldrich). After incubation, cells were fixed (Perm A; Life Tech) and stained using anti-CD16 allophycocyanin (APC)-Cy7 (BD), anti-CD56 PE-Cy7 (BD), and anti-CD3 Pacific Blue (BD). Intracellular staining using anti-gamma interferon (IFN-γ) FITC (BD) and anti-MIP-1β PE (BD) was performed after permeabilizing the NK cells using Perm B (Thermo Fisher). Flow cytometry acquisition of all assays was performed using an iQue (Intellicyt) and an S-LAB robot (PAA). For ADCP, phagocytosis events were gated on bead-positive cells. For ADNP, neutrophils were identified by gating on CD3^−^, CD14^−^, and CD66b^+^ cells. Neutrophil phagocytosis was identified by gating on bead-positive cells. A phagocytosis score for ADCP and ADNP was calculated as (percentage of bead-positive cells) × (mean fluorescent intensity [MFI] of bead-positive cells) divided by 10,000. ADCD quantification was reported as the MFI of FITC-anti-C3. For antibody-dependent NK activation, cells were identified by gating on CD3^−^, CD16^+^, and CD56^+^ cells. Data were reported as the percentage of cells positive for CD107a, IFN-γ, and MIP-1β.

### Viral genome amplification and sequencing.

Viral RNA was extracted from nasopharyngeal specimens utilizing the QIAamp viral RNA minikit (Qiagen). cDNA synthesis was performed with a SuperScript IV first-strand synthesis kit (Thermo) using random hexamer primers according to the manufacturer’s specifications. A multiplex primer sequence set, comprised of two nonoverlapping primer pools, was created using Primal Scheme and provided by the Artic Network (v3). Amplification of the viral genome cDNA was performed in multiplexed PCRs to generate ∼400-bp tiled amplicons across the genome. PCR amplification was carried out using Q5 Hot Start HF *Taq* polymerase (NEB) and validated by agarose gel electrophoresis.

Amplicons from both primer pools were combined and purified with a 1× volume of AmpureXP beads (Beckman Coulter). DNA was treated with KAPA HyperPrep end prep enzyme mix (KAPA prior to barcoding with NEXTflex barcodes and KAPA HyperPrep DNA ligase) for simultaneous sequencing. Samples were pooled and purified with a 0.8× volume of AmpureXP beads, and library amplification was performed using KAPA HiFi HotStart with KAPA library amp primers. Amplicons were purified with a 0.8× volume of AmpureXP beads, normalized to 5 nM, and pooled. The pooled library was denatured and loaded onto a MiSeq v2 500 cycle flow cell (Illumina). Viral genome consensus sequences were determined from sequencing reads as previously described (PMID 30621750) (43). Sequencing reads were aligned to the reference SARS-CoV-2 genome sequence MN908947.3 using bwa version 0.7.15. Pileups were generated from the alignment using SAMtools v1.9. Barcode sequences were trimmed from aligned reads and consensus sequence determined using iVar v1.2.2 (PMID 30621750) (43) using a minimum alignment depth of 10 reads, a minimum base quality of 20, and a consensus frequency threshold of 0 (i.e., majority base as the consensus). Consensus sequences with ≥10% missing bases were discarded. Pango lineages were assigned to consensus sequences using pangolin software (PMID 32669681; https://github.com/cov-lineages/pangolin) (44).

### Phylogenetic analysis.

Consensus sequences obtained from the patient and 500 randomly selected sequences from the GISAID database uploaded before 18 February 2021 were aligned using MAFFT v7.453 software and manually edited using MEGA v6.06. Using these aligned sequences, we inferred a maximum likelihood (ML) phylogeny with IQ-Tree v2.0.5 using its ModelFinder function to estimate the nucleotide substitution model best fitted for the data set by means of Bayesian information criterion (best-fit model, GTR + F + R2). We assessed the tree topology for each phylogeny both with the Shimodaira-Hasegawa approximate likelihood-ratio test (SH-aLRT) and with ultrafast bootstrap (UFboot) with 1,000 replicates each. The two patient sequences clustered in different lineages of the tree with strong statistical support (SH-aLRT > 90 and UFboot =100).

### Analysis.

Seropositive individuals were identified through a community-based serosurveillance program, wherein ELISA was performed to detect IgG against the RBD of the SARS-CoV-2 spike glycoprotein in 4,469 subjects. A concentration-based cutoff was established to determine positivity as a concentration value five standard deviations above the mean micrograms per milliliter in negative-control samples. This assay performance has previously been externally validated in a blinded study as 99.6% specific (45).

The Wilcoxon signed-rank test was used to identify the significance of antibody feature changes between week 5 and week 6 in macaques and R-1 and R0 in humans. The Python package statsmodel (version 0.11.1) (46) was used to adjust the *P* values generated from the Wilcoxon test, filtering using the Benjamini/Yekutieli method for a false discovery rate of 5%.

Individuals were marked as potentially reinfected if they were tested as PCR positive more than 15 days after an initial seropositive result. Eleven individuals meeting these criteria were identified, out of which we had access to serum samples post-PCR testing for nine. Out of these nine, six individuals had a PCR^+^ result more than 40 days after initial seropositivity, with all but one individual testing positive more than 25 days after seropositivity. System serology was then performed for these nine individuals.

Two types of one-antibody feature models were generated, (i) using a simple threshold/cutoff to identify reinfections status based on one immunological feature from one serum sample and (ii) using the relative difference between Luminex titer in subsequent weeks to assess a change of one feature between two time points. Differences in Luminex titer between consecutive weeks were computed based on raw titer for use in univariate classifier generation. Thresholds were identified by maximizing the difference between the true-positive rate and false-positive rate. We note that due to the simple nature of these predictors, thresholds were trained on all available samples, with all applicable predictors tabulated and available as supplemental tables. Models containing two antibody features were constructed as delta models, where the relative difference between two time points was computed. Models trained and tested on humans were trained using logistic regression and cross-validated with 4-fold cross validation, along with an 80% to 20% split of training/cross validation and test sets. All chronologically ordered pairs of serum samples were assembled to define the training set. For example, given primate serum samples for week 2, week 5, week 6, and week 7, {week 2, week 5}, {week 6, week 7} were labeled not associated with reinfection, whereas {week 2, week 6}, {week 2, week 7}, {week 5, week 6}, and {week 5, week 7} were labeled associated with reinfection. We note that for both primates and humans, serum samples prior to the first identified infection event were not included in the training set, so as to constrain our model classification to identifying reinfection given primary infection compared to the situation without *a priori* information. Classification accuracies were then computed as the percentage of correct classifications. For cross-species analysis, logistic regression models were trained using all available primate data and cross-validated and tested in humans with a 50-50 split in data. We note that our primate assays included FcγR2A subtypes 1, 2, 3, and 4, whereas our human assays were pan-FcγR2A. As such, cross-species models trained on FcγR2A subtypes were directly mapped to FcγR2A parameter values in humans. Finally, we note that due to the similarity of performance of various models in the cross validation steps, we opted to report test set performance on numerous (50) high-performing models from the cross validation step as an alternative to implying unique importance of the features of only the best-performing model. As a final validation step, we compared our reported logistic regression models to those created by different random initialization states to ensure that reported results were consistent with results from different states.

Bar graphs, *x-y* plots, and heatmaps were generated using Python version 3.7.3 (Python Software Foundation; www.python.org). Study overview diagrams were generated in part using BioRender, a cloud-based platform for figure design. Principal-component analysis, logistic regression, and receiver operating characteristic curves were performed using scikit-learn version 0.23.2 (47).

10.1128/mBio.02141-21.10 DATA SET S1 Excel file containing patient and macaque antibody data used in this study. Sheet 1, sequencing-confirmed patient, Luminex data from sequencing-confirmed patient at different timepoints of infection. Sheet 2, ELISA, ELISA data from SpaceX cohort used in this study. Sheet 3, Luminex, Luminex data from SpaceX cohort used in this study. Sheet 4, monkey_data, Luminex data from macaque cohort used in this study. Download Data Set S1, XLSX file, 0.6 MB. Copyright © 2022 Siddiqui et al. 2022 Siddiqui et al. https://creativecommons.org/licenses/by/4.0/ This content is distributed under the terms of the Creative Commons Attribution 4.0 International license.
